# Teaching and learning socio-emotional skills and their influence on interprofessional work

**DOI:** 10.1590/0034-7167-2025-0295

**Published:** 2026-11-06

**Authors:** Laura Andrian Leal, Jaqueline Alcântara Marcelino da Silva, Iasmin Gabrielli Silva, Carolina Cassiano, Fabiana Faleiros, Aline Teixeira Silva, Ivaneia Alves Pereira, Silvia Helena Henriques

**Affiliations:** I Universidade de São Paulo. Ribeirão Preto, São Paulo, Brazil; II Universidade Federal de São Carlos. São Carlos, São Paulo, Brazil; III Universidade do Estado de Minas Gerais. Passos, Minas Gerais, Brazil

**Keywords:** Nurses, Education, Nursing, Professional Competence, Emotions, Interprofessional Relations., Enfermeros, Educación en Enfermería, Competencia Profesional, Emociones, Relaciones Interprofesionales.

## Abstract

**Objectives:**

to analyze nursing undergraduate students’ perceptions regarding teaching-learning strategies for socio-emotional skills and their influence on interprofessional work.

**Methods:**

a qualitative, exploratory, and descriptive study conducted with 57 nursing undergraduate students from a university in the countryside of the state of São Paulo. In 2023, focus groups and inductive thematic analysis were employed.

**Results:**

undergraduate students reported that the topic is not explored in depth during their education, although they recognized the strong influence of socio-emotional skills on interprofessional work, favoring team effectiveness, integration, and productivity. They emphasized the importance of effective communication, conflict resolution, resilience, empathy, and mutual understanding for collaborative work.

**Conclusions:**

investing in the development of these skills contributes to improving interaction among team members and the outcomes achieved. Reflecting on this gap may encourage educational institutions to take action toward including these skills in professional education.

## RESUMEN

**Objetivos:**

analizar la percepción de los estudiantes de enfermería de pregrado sobre las estrategias de enseñanza-aprendizaje de habilidades socioemocionales y su influencia en el trabajo interprofesional.

**Métodos:**

se realizó un estudio cualitativo, exploratorio y descriptivo con 57 estudiantes de enfermería de pregrado de una universidad del interior del estado de São Paulo. En 2023, se utilizaron grupos focales y análisis temático inductivo.

**Resultados:**

los estudiantes de pregrado indicaron que el tema no se explora en profundidad durante sus estudios de pregrado, aunque reconocen la fuerte influencia de las habilidades socioemocionales en el trabajo interprofesional, lo que favorece la eficacia, la integración y la productividad de los equipos. Subrayaron la importancia de la comunicación efectiva, la resolución de conflictos, la resiliencia, la empatía y la comprensión mutua para el trabajo colaborativo.

**Conclusiones:**

invertir en el desarrollo de estas habilidades contribuye a mejorar la interacción entre los miembros del equipo y los resultados obtenidos. Reflexionar sobre esta brecha puede incentivar a las instituciones de formación a incluirlas en el desarrollo profesional.

## INTRODUCTION

Nursing, as a profession directly focused on the care and well-being of healthcare service users, requires professionals to work collaboratively and possess not only mastery of technical procedures, but also a solid foundation of socio-emotional skills (SESs), which are essential for effective collaborative practice. The importance of nurses’ collaborative involvement with other professionals to provide high-quality care is not a recent topic, having long been recognized in governmental agendas and nursing practice guidelines throughout the Western world^(1-4)^. Thus, considering the various activities performed by nurses, a training process operationalized through dimensions such as SESs is vital in order to contribute to education that encompasses emotional management and collaboration among different professional categories.

In this context, the definition of competence is polysemic, being predominantly defined by knowledge, skills, attitudes, and values, and is mainly related to the mobilization of capacities to respond to complex social and professional demands^(5)^. In this study, SESs are conceptualized as behavioral capacities learned by individuals in response to the demands of situations involving social interaction, including communication, problem-solving, cooperation, empathy, and assertiveness. It is important to emphasize that emotional competence can be learned and is grounded in emotional intelligence. Thus, it is observed that individuals who possess emotional intelligence apply SESs^(6)^.

This dimension is directly articulated with interprofessional competencies, understood as those developed by professionals from different fields with the aim of improving teamwork and the quality of care, especially in the healthcare field. Therefore, interprofessional work leads to collaborative practices. Unlike specific competencies (particular to each profession) and common competencies (the intersection among them), collaborative competencies promote mutual learning and sustain interprofessional work^(7,8)^. In this regard, SESs, by fostering empathy, cooperation, and assertiveness, constitute the behavioral and emotional foundation necessary for the implementation of collaborative competencies, becoming indispensable for integrated professional practices centered on the quality of interaction and care. This understanding broadens the concept of competence beyond technical mastery by incorporating relational, ethical, and communicational dimensions^(6,9)^.

Within the field of interprofessionality, classical frameworks highlight that collaborative practice requires specific competencies for teamwork^(9-11)^. The Canadian Interprofessional Health Collaborative (CIHC) proposes a competency framework that includes interprofessional communication, teamwork, collaborative leadership, and patient-centered care. Such competencies are fundamental for complex healthcare settings such as those experienced in nursing^(10)^.

Recognizing the existence of different consolidated conceptual frameworks in the field of interprofessionality, such as those proposed by Barr, the Interprofessional Education Collaborative, and the Australian framework, this study primarily engages with the CIHC framework due to its relevance to the educational and healthcare context of nursing^(7-11)^.

The integration between SESs and interprofessional work proves indispensable, since collaboration transcends merely technical or organizational dimensions and is intrinsically linked to relational and emotional processes that ensure the effectiveness of interactions. Without SESs, interprofessional work tends to become superficial, marked by conflicts and communication failures; on the other hand, SESs alone lack collective impact. The articulation between both enables the overcoming of fragmented models of practice, promoting interdependence, mutual learning, and ethical and humanized environments. In the healthcare field, this convergence is decisive for ensuring patient safety and the effectiveness of interprofessional practices, configuring itself as a structuring axis of contemporary professional education and practice^(12,13)^.

Currently, many nurses work in interprofessional teams^(4,14,15)^, making collaboration increasingly relevant, with nursing being the profession that most contributes to articulation among professionals due to the nature of its work centered on patient care^(16)^. In this sense, nursing education has been supported by the Brazilian National Curriculum Guidelines (DCNs - *Diretrizes Curriculares Nacionais*), which present guiding competencies for professional education, such as leadership, teamwork, among others. The most recent version, approved by Resolution CNE/CES 573/2018, reinforces the importance of critical, ethical, humanistic, and health-committed education^(17,18)^. Therefore, it becomes essential to articulate education in favor of interprofessionality.

As previously discussed, nurses are considered professionals who coordinate care, being predominantly the category that remains in continuous contact with healthcare service users, especially in hospital and inpatient settings, where they are available 24 hours a day. Thus, the use of collaborative practices by this category becomes essential for promoting safe and high-quality actions, although other professionals also play relevant roles in this process. The presence of interprofessionality in the nursing DCNs highlights the need to overcome fragmented models of education and practice. By positioning collaboration among professionals as a structuring axis, DCNs aim to educate nurses who are better prepared for teamwork, the construction of shared therapeutic plans, and the continuous improvement of healthcare quality^(15,19)^.

The framework used in the field of interprofessionality is psychological safety, as it allows team members to feel emotionally safe to participate fully and collaborate effectively. Thus, although team effectiveness has been shown to improve when colleagues feel emotionally safe with one another^(20)^, few interprofessional initiatives address the dynamics of SES development in education for successful collaborative practices, especially in Brazil. Furthermore, although another study does not explicitly use the term “psychological safety factor”, the author discusses aspects related to the culture of the clinical environment, trust in relationships, and support from preceptors, which approximate the idea of psychological safety (for example: feeling welcomed, being able to communicate without fear, and trusting colleagues)^(21)^.

Thus, this study presents the following research question: what are nursing undergraduate students’ perceptions regarding how the teaching-learning of SESs may impact interprofessional work?

The need to investigate nursing undergraduate students’ perceptions regarding the teaching-learning of SESs and their impact on interprofessional work lies in the fact that, although these capacities are recognized as important, their effective practice is not always adequately cultivated in traditional educational curricula. It is observed that educational programs focus on the development of specific technical capacities while relegating the promotion of SESs for interprofessional practice to the background. By addressing this gap, the present study may stimulate reflections by educational institutions regarding the creation of strategies integrating the development of SESs.

Therefore, by addressing this theme, the research may contribute to advancing nursing participation in the dynamics of interprofessional teams, demonstrating that investing in the development of SESs can not only improve interaction among team members, but also optimize the outcomes achieved.

## OBJECTIVES

To analyze nursing undergraduate students’ perceptions regarding teaching-learning strategies for SESs and their influence on interprofessional work.

## METHODS

### Ethical aspects

The study received approval from the Research Ethics Committee of the proposing institution, in accordance with Resolution 466/12. Participants signed the Informed Consent Form, which ensured the confidentiality of the information provided.

### Study design

This is an exploratory and descriptive study using a qualitative approach for data collection. Qualitative research, in this case, is appropriate because it enables the investigation of experiences, perceptions, behaviors, cultures, and social relationships, allowing a holistic and detailed understanding of the realities experienced by participants^(22)^. The article was prepared in accordance with the COnsolidated criteria for REporting Qualitative research guidelines^(23)^.

### Study setting

The research was conducted at a Brazilian public university within the nursing program, which offers two distinct undergraduate programs: bachelor’s degree in nursing and bachelor’s degree and teaching credential in nursing. On average, the institution graduates 130 students per year and currently has a total of 565 undergraduate students enrolled across all academic years.

### Data source

Study participants were final-year undergraduate students from the aforementioned programs who had completed the course entitled “Hospital Nursing Management”. These undergraduate students were included due to the initial knowledge already explored during their education regarding competencies, emotions, and experience, considering that at this stage they had already completed internships focused on administration and management, emphasizing the skills necessary for nursing practice. Undergraduate students who had not completed the listed course, who had suspended enrollment, or who were on leave were excluded from the study.

### Data collection and organization

Data collection took place between March and September 2023. Data were obtained through the focus group (FG) technique, which involved the following stages: the first consisted of a sociodemographic questionnaire including information on gender, origin (place of birth), and type of undergraduate program; the second used a script with guiding questions for group discussion, in accordance with the methodological framework described by Gatti^(24)^.

A non-probabilistic purposive sampling method was employed. Participants were formally invited through an invitation letter distributed after class or sent electronically, allowing them to schedule research meetings according to their convenience.

Nine FGs were conducted, with eight groups composed of six undergraduate students and one group composed of nine undergraduate students. All groups were conducted in person and allowed for random grouping of participants. Data collection was moderated by a researcher with a doctoral degree in sciences and supported by an observer, a nurse with a master’s degree in psychology and a doctoral degree in sciences. Both the moderator and observer were affiliated with the same educational institution but did not act as instructors, which helped mitigate any potential influence of authority.

FG meetings were audio-recorded and lasted an average of 60 minutes. The guiding questions for the discussions were: have you had any course or content addressing SESs? And interprofessional work? What are your perceptions regarding the influence of SESs on interprofessional work? Do you perceive that the university implemented strategies to develop these competencies in you? The questions were developed by the principal researcher, and a field diary was used to document perceptions during and after the FGs, which supported data analysis. Data collection was considered complete when information saturation was reached.

### Data analysis

For data analysis, group discussions were manually transcribed by the principal researcher without the aid of software and analyzed using the inductive thematic analysis technique, supported by the theoretical framework of interprofessionality and SESs^(6,10)^. This process involved transcription, coding, topic grouping, detail refinement, and final analysis^(25)^. This type of analysis is considered ideal because it seeks to extract meanings from participants. To maintain anonymity, participants’ statements were identified by FG number (FG1, FG2, etc.), and quotations were structured as follows (FG1. Student 1...), ensuring that the identities of all individuals involved remained confidential.

## RESULTS

A total of 57 participants answered the questionnaire, most of whom were undergraduate students: 51 (89.47%) were biologically female and six (10.52%) male. Concerning the undergraduate program, 41 (71.92%) were enrolled in the bachelor’s degree in nursing program, and 16 (28.07%) in the bachelor’s degree and teaching credential program. Regarding place of birth, most participants were from the state of São Paulo (96%), while the remaining participants were from the state of Minas Gerais (4%). Based on the analysis of the findings, two categories were constructed: “Socio-emotional skills, interprofessional work, and teaching-learning”; and “Influence of socio-emotional skills on interprofessional work”.

### Socio-emotional skills, interprofessional work, and teaching-learning

For the undergraduate students, SESs are associated with teamwork, shared leadership, professional attitude, visibility, adaptability, among others:

*For me, socio-emotional skills are about posture, ethics, separating the personal from the professional. And when we have that, we can adapt to the team, to the sector, and be recognized, you know? It gives visibility.* (FG 1. Student 3)

*I see today, after many internships, that, of course, nursing doesn’t work alone, but to achieve that, you need these skills: clear communication, respect, sharing decisions, being a leader who accepts listening to and heeding others.* (FG 2. Student 4)

Regarding interprofessional work, the undergraduate students associated the definition with the term “multidisciplinary” and highlighted the challenges of putting it into practice during internships:

*Look, I’ve heard of interprofessional, but we learned about multidisciplinary, and from what I understand, it’s different, right?! But the problem is that, even having the opportunity to do various types of internships in various places, I still haven’t seen this collaboration. It still largely depends on the doctor’s decision.* (FG 5. Student 9)

*We learned, or rather, concluded during the internship that the work happens in a team, it needs to involve multidisciplinary teams, otherwise the patient is not treated holistically. Therefore, several professions must be involved.* (FG 7. Student 6)

Despite the importance of SESs and interprofessional work, within the educational context the development of these competencies faces several challenges. For some undergraduate students, SESs are still viewed as “additional” or secondary in relation to academic content, which hinders their integration into educational institutions.

*Unfortunately, that part about socio-emotional skills that they mentioned wasn’t taught. We had a lot of content about humanization, communication, but with the patient* [...] *nothing geared towards us as nurses, you know? We even had a class on how to communicate bad news and death, but again, it was geared towards the patient.* (FG 4. Student 1)

*I see that the university wants to prepare the perfect robot nurse for the patient, but this thing about socio-emotional skills is secondary. We even talk about teamwork, but in practice, it’s each profession for itself, because first and foremost are the procedures and the patient’s well-being.* (FG 5. Student 8)

### Influence of socio-emotional skills on interprofessional work

SESs exert a strong influence on interprofessional work, playing a fundamental role in the effectiveness, harmony, and productivity of teams composed of professionals from different fields. When well developed, these skills significantly contribute to the success of interprofessional teams, both regarding the quality of the work performed and the well-being of professionals and individuals:

*Look, at the hospital, it was more difficult for me to see integration and collaboration, but at BHU, the team was very united, you know? And they decided everything together, and I realized, observing, how good that was for care, for the patients, you know?* (FG 6. Student 2)

*I think that when a person has the ability to control their emotions and understand being empathetic with others, they can work better for the benefit of patients.* (FG 9. Student 6)

*If we respect each other, we can work better together, and that can help with well-being, because what I saw most was conflict and lack of education, which is why the work didn’t happen in an integrated way.* (FG 7. Student 3)

At the same time, according to the undergraduate students, in order to work in an interprofessional or collaborative manner, professionals must know how to communicate effectively, resolve conflicts, manage stress, develop resilience, empathy, and mutual understanding, which are SESs themselves:

*I realized that, for example, here at the hospital, of course there’s teamwork, but for it to work, you need to respect, be empathetic, and ethical. And dealing with people is difficult, but for the team to function, you have to respect each other.* (FG 2. Student 5)

*People are difficult, aren’t they? I witnessed many conflicts in the sector, a lot of arguing, and it was common for one person not to talk to the other and not to ask for help just because they didn’t want to be with their colleague, and that hinders care, right? Everyone was stressed, so I see that stress management is important.* (FG 1. Student 4)

*The nurse, by having this socio-emotional skill to work, requires flexibility and the ability to adapt to new situations and, of course, also to understand the other person.* (FG 5. Student 5)

*Even though here at the hospital it’s a bit fragmented, as people don’t talk to each other much and the decision is mostly made by the doctor, I see that it’s necessary to have emotional control, you know?* (FG 8. Student 1)

Therefore, this influence is linked to the good examples set by educational leaders in internship settings and teaching practice, who contributed to the development and application of SESs in interprofessional work. The undergraduate students stated that these leaders should not only serve as models of emotionally intelligent behavior, but also work to cultivate these skills within their teams, since they create a work environment in which empathy, open communication, and collaboration are everyday practices fundamental to team success. In addition, they serve as models of emotional behavior, establish safe spaces for dialogue, promote professional development, and recognize diversity:

*At the hospital where I am, there is a wonderful nurse who fits this question. I think being emotionally prepared has everything to do with collaborative work, because this nurse is an example of how to deal with situations of pressure and conflict. She speaks well, you know? Clear and she is firm, but always opens doors for technicians to learn. She encourages people to improve themselves, so I saw that she creates a culture of support and trust, not only for the technicians, but even the doctors ask for her opinion and respect it, physiotherapists, etc.* (FG 3. Student 4)

*I saw this a little more at the BHU where I did my internship, because the nurse listened and seemed to validate these experiences, which I saw, and I think it leads to better solutions for the clinical case and greater unity in the group.* (FG 1. Student 4)

That said, interprofessional work is not always easy to implement in practice. Lack of time, innovative methodologies, resources, and adequate educational planning often prevent undergraduate students from clearly understanding the importance of working collaboratively and in an integrated manner. The division of work among different areas of knowledge and the hierarchical organization of professions may also hinder the construction of a truly collaborative environment:

*During my internship, I hardly saw any interprofessional work involved. Everyone was overloaded; the nurse didn’t have time to foster a culture of teamwork and exchange, you know? It was just a matter of giving orders and that was it. Besides, the doctors weren’t trained for that either. They think they’re in charge.* (FG 6. Student 2)

*I hardly had any discussion of this during my undergraduate studies. There were occasional lectures from leagues or scientific initiation projects, but in class, we didn’t have a clear understanding of how to behave and interact with other people*. (FG 7. Student 2)

*I discussed this during my internship with my professor* [...] *she asked me how the team solved it, but I only saw this from one professor, not in the other internships.* (FG 4. Student 4)

*I’ll try to find out about this individually. I didn’t see it during my undergraduate studies, but it’s difficult because I’m someone who speaks up, but there are some professionals who are difficult and say, “That’s my area”. Or that the doctor decides the final outcome.* (FG 3. Student 3)

## DISCUSSION

The results showed that undergraduate students associated SESs with teamwork, shared leadership, ethical conduct, and adaptability, although they pointed out difficulties in understanding and practicing interprofessional work, which was frequently confused with multidisciplinary work and marked by physician-centeredness. They also highlighted the limited integration of SESs into academic education and emphasized the influence of these competencies on the effectiveness and harmony of interprofessional teams, stressing skills such as empathy, communication, conflict management, and emotional control as indispensable for the quality of care and the well-being of professionals.

Furthermore, they also attributed relevance to the role of educational leaders as models of emotional intelligence capable of promoting collaborative environments. However, they identified structural and cultural barriers, such as professional hierarchization, work overload, and the absence of pedagogical methodologies, which hinder the consolidation of interprofessional work. As observed in the study, SESs are not explored in depth within the academic context, although they are intended to support collaborative practices.

It is observed that universities often prioritize what can be easily taught, tested, and validated within their traditional methodologies, mainly based on examinations, clinical assessments, and technical knowledge. This results in curricula that favor the learning of skills directly related to healthcare service users’ care, such as clinical practices and diagnosis^(26)^. Another important point is that SESs are viewed as something professionals supposedly develop through experience and lived practice. This perspective may lead to failures in recognizing that these skills can and should be taught, trained, and assessed more systematically within the academic environment^(27)^.

Another relevant factor is that academic education has traditionally focused on developing knowledge, often in isolation, without a clear emphasis on integration among different fields of knowledge. In healthcare fields, programs frequently approach education by preparing professionals to work individually rather than preparing students for collaborative work within interprofessional teams^(28,29)^, environments in which SESs are essential.

Moreover, the increasing pressure for universities to educate highly specialized professionals capable of dealing with complex technical issues has also led to a mismatch with the need for more holistic education. In this line of thought, it is evident that universities offer education based on disciplines and specialties. In many fields of knowledge, programs are organized to prepare students to work within a single area of specialization, emphasizing the development of technical and theoretical competencies related to their specific field^(28,30)^.

Thus, it is observed that the academic curriculum in many institutions remains structured according to the logic of training professionals as “specialists” restricted to their own field of practice, rather than preparing them to become active collaborators within interprofessional teams. This limited perspective is reflected in the study findings, in which undergraduate students demonstrated confusion between the concepts of interprofessional and multidisciplinary work, evidencing the absence of education that values integration, cooperation, and the collective construction of care.

University infrastructure may also be a limiting factor. Many institutions do not have sufficient resources to effectively promote interprofessional teaching practices. However, the literature reveals that practical activities and real interprofessional experiences are possible, such as joint internships, team projects, and interprofessional simulations, which foster efficiency among different departments and programs^(31,32)^. This requires substantial investment in time, resources, and institutional planning, something that is not always feasible in academic environments facing budgetary or structural constraints.

Nevertheless, researchers add that faculty members’ lack of knowledge regarding the topic, or even the time and creativity required, may compromise the development of these competencies among nursing undergraduate students^(33)^. However, there are reports of successful international experiences. As an example, authors described an interprofessional simulation case involving a standardized patient, in which medical and social work students worked together using motivational interviewing. The results indicated that undergraduate students learned how different professions approach health-related issues and communicate to patients the importance of collaboration and the need for patient-centered care as an integrated care approach. Thus, these findings suggest that standardized interprofessional simulations may contribute to effective, comprehensive, and collaborative learning^(34)^.

At the same time, these gaps in teaching about interprofessional work culminate in failures in healthcare delivery, as well as confusion between the terms “interprofessional” and “multidisciplinary”, as observed in the findings. In the multidisciplinary model, professionals from different fields work together, but their practices tend to be more independent. Although each professional is part of the team, they perform their tasks in isolation, respecting their own knowledge and limits of practice. Interaction among team members may occur, but it is generally less integrated, with each professional focused on their own area of specialization. In interprofessional work, however, collaboration is the central element. Professionals from different fields work in a more integrated way, sharing knowledge, discussing cases, and making decisions together^(35)^.

Thus, the influence of SESs on interprofessional work becomes evident, directly impacting effectiveness, harmony within the work process, and, above all, comprehensive and high-quality care for healthcare service users^(15,36)^. Regarding this reflection, one of the most evident forms of influence occurs through communication. When professionals are able to express their ideas clearly and assertively, while remaining respectful and empathetic, the exchange of information becomes more efficient and mutual understanding is facilitated. This reduces the possibility of misunderstandings and facilitates collective decision-making, which is essential for achieving common goals^(37)^. This is particularly useful in educational environments, making the development of various communication-focused educational strategies for the entire healthcare team indispensable.

Furthermore, leadership in interprofessional teams is significantly strengthened through the development of SESs. As observed in our study, the figure of the professor or preceptor with active and collaborative leadership, grounded in active listening, mutual support, and appreciation of all team members, proves more effective than authoritarian or professionally hierarchical models. Unlike verticalized approaches centered on position or profession, collaborative leadership promotes environments of trust, encourages shared responsibility, and enhances integration among different fields of knowledge, aspects that are indispensable for consolidating interprofessional work. Moreover, good leaders and positive role models play a fundamental role in the success of interprofessional work because they help create an environment conducive to collaboration, mutual respect, and effectiveness^(38)^.

Consequently, SESs are essential for the success of interprofessional work because they help create an environment of trust, collaboration, and mutual respect. As demonstrated in both our theoretical framework and findings, SESs not only improve communication and conflict resolution, but also promote empathy, resilience, and adaptability, all of which are fundamental for the effective functioning of teams composed of professionals from different fields. Therefore, developing these skills is an investment that benefits everyone involved, creating a more harmonious and productive environment^(12,13)^, which should be reflected upon and incorporated into pedagogical discussions within educational programs.

Despite the academic gap regarding the in-depth exploration of SESs and interprofessional work, there is a growing movement within universities and educational institutions to integrate these competencies into academic curricula. Many fields have already formalized the understanding that a good professional is not only one who possesses technical knowledge, but also one who knows how to relate to, communicate with, and collaborate with others, whether colleagues or healthcare service users. More humanized educational programs, including topics such as emotional intelligence, assertive communication, conflict resolution, and teamwork, are beginning to be incorporated, especially in healthcare and education contexts^(39)^. However, this change remains gradual and depends on greater awareness of the importance of these skills for professional performance.

Thus, the findings reinforce the need to systematically integrate SESs into the educational process, articulating them with collaborative competencies as a structuring axis of healthcare education and practice. In this way, creating a balanced and welcoming classroom environment conducive to the development of values and the collaborative construction of disciplinary knowledge has become an increasingly greater challenge. To achieve this, professors must mobilize a broad range of competencies, such as SESs^(40)^.

### Study limitations

This research presents the limitation of having been conducted exclusively at a public university, without including analyses from other educational institutions. In addition, the study focused solely on final-year nursing undergraduate students. Therefore, it is essential to emphasize the need for future studies encompassing other educational institutions, including private ones, as well as involving various professionals and educators. This will help determine whether discrepancies exist in the findings and to what extent they may be generalized.

### Contributions to nursing, health, or public policies

By investigating how undergraduate students perceive the approaches used to develop competencies such as empathy, communication, collaboration, emotional self-regulation, and active listening, the study provides relevant support for improving undergraduate curricula, aligning them with the contemporary demands of healthcare, which increasingly require effective interprofessional practice.

Furthermore, by directly relating the development of SESs to teamwork practice among different professional categories, the study contributes to strengthening collaborative practice within the context of the Brazilian Unified Health System and other healthcare settings. Valuing undergraduate students’ perspectives also favors the construction of more student-centered pedagogical strategies, promoting student protagonism in the learning process and the development of more human, critical, and reflective education. Thus, the study dialogues with national health education guidelines, such as the DCNs for undergraduate nursing programs, by proposing ways to integrate technical-scientific education with the ethical and relational education of future professionals.

## FINAL CONSIDERATIONS

This study demonstrated that, in nursing undergraduate students’ perceptions, SESs are necessary for interprofessional work because they directly influence communication, conflict resolution, empathy, mutual understanding, and integration among healthcare professionals. Thus, the research question is answered by demonstrating that the teaching-learning of these competencies significantly impacts the effectiveness and quality of collaborative work.

In line with the study objective, the results indicated that although undergraduate students recognize the relevance of SESs for interprofessional practice, opportunities for developing these competencies during undergraduate education were scarce and minimally present within the formal curriculum, occurring in isolated and non-systematic ways.

Therefore, the findings reinforce the need for educational institutions to intentionally and systematically incorporate pedagogical strategies aimed at developing SESs in order to prepare nurses who are better equipped for interprofessional work and the contemporary demands of healthcare.

## Data Availability

The research data are available within the article.
