# Supplementary material for: Teaching and learning socio-emotional skills and their influence on interprofessional work
Source: Rev Bras Enferm. 2026 Nov 6;79:e20250295. doi: 10.1590/0034-7167-2025-0295 (PMC13742973; doi:10.1590/0034-7167-2025-0295)
Supplement: Translation

# Translation: Ensino-aprendizagem de competências socioemocionais e sua influência no trabalho interprofissional

**DOI:** 10.1590/0034-7167-2025-0295pt

**Authors:** Laura Andrian Leal, Jaqueline Alcântara Marcelino da Silva, Iasmin Gabrielli Silva, Carolina Cassiano, Fabiana Faleiros, Aline Teixeira Silva, Ivaneia Alves Pereira, Silvia Helena Henriques

**Affiliations:** I Universidade de São Paulo. Ribeirão Preto, São Paulo, Brasil; II Universidade Federal de São Carlos. São Carlos, São Paulo, Brasil; III Universidade do Estado de Minas Gerais. Passos, Minas Gerais, Brasil

**Keywords:** Enfermeiros, Educação em Enfermagem, Competência Profissional, Emoções, Relações Interprofissionais.

## Abstract

**Objetivos:**

analisar a percepção de graduandos de enfermagem sobre estratégias de ensino-aprendizagem de competências socioemocionais e sua influência no trabalho interprofissional.

**Métodos:**

estudo qualitativo, exploratório e descritivo, com 57 graduandos de enfermagem de uma universidade do interior paulista. Em 2023, foram utilizados o grupo focal e a análise temática indutiva.

**Resultados:**

os graduandos elencaram que a temática não é aprofundada na graduação, embora reconheçam a forte influência das competências socioemocionais no trabalho interprofissional, favorecendo a eficácia, a integração e a produtividade das equipes. Ressaltaram a importância da comunicação eficaz, a resolução de conflitos, a resiliência, a empatia e a compreensão mútua para o trabalho colaborativo.

**Conclusões:**

investir no desenvolvimento dessas competências contribui para melhorar a interação entre os membros da equipe e os resultados alcançados. Refletir sobre essa lacuna pode impulsionar ações por parte das instituições formadoras para sua inclusão na formação profissional.

## INTRODUÇÃO

A enfermagem, por ser uma profissão diretamente voltada ao cuidado e ao bem-estar dos usuários dos serviços de saúde, exige que os profissionais atuem de forma conjunta e possuam não apenas domínio de procedimentos técnicos, mas também uma sólida base de competências socioemocionais (CSEs), essenciais para uma prática colaborativa eficaz. A importância do envolvimento colaborativo dos enfermeiros com outros profissionais para fornecer cuidados de alta qualidade não é tema recente, sendo reconhecido nas agendas governamentais e nas diretrizes de prática da enfermagem em todo o mundo ocidental^(1-4)^. Assim, diante das diversas atividades que o enfermeiro realiza, é vital um processo formativo operacionalizado em vertentes, como as CSEs, a fim de contribuir para uma formação que contemple o manejo das emoções e a colaboração entre diferentes categorias profissionais.

Nesse contexto, a definição de competência é polissêmica, sendo majoritariamente definida por conhecimentos, habilidades, atitudes e valores, e relaciona-se sobretudo à mobilização de capacidades para responder às complexas demandas sociais e profissionais^(5)^. Neste estudo, adota-se como conceito de CSE as capacidades comportamentais aprendidas pelo indivíduo diante das demandas de uma situação de interação social, incluindo comunicação, resolução de problemas, cooperação, empatia e assertividade. Vale salientar que a competência emocional é passível de ser aprendida e é pautada na inteligência emocional. Assim, observa-se que pessoas que possuem inteligência emocional aplicam as CSEs^(6)^.

Essa dimensão se articula diretamente com as competências interprofissionais, entendidas como aquelas desenvolvidas por profissionais de diferentes áreas com vistas à melhoria do trabalho em equipe e da qualidade do cuidado, sobretudo no campo da saúde. Assim, o trabalho interprofissional acarreta práticas colaborativas. Diferenciando-se das competências específicas (próprias de cada profissão) e das comuns (interseção entre elas), as competências colaborativas promovem o aprendizado mútuo e sustentam o trabalho interprofissional^(7,8)^. Nesse interim, observa-se que as CSEs, ao favorecerem a empatia, a cooperação e a assertividade, constituem a base comportamental e emocional necessária para a efetivação das competências colaborativas, tornando-se indispensáveis para práticas profissionais integradas e centradas na qualidade da interação e do cuidado. Essa compreensão amplia o entendimento da competência para além do domínio técnico, incorporando dimensões relacionais, éticas e comunicacionais^(6,9)^.

No âmbito da interprofissionalidade, referenciais clássicos destacam que a prática colaborativa requer competências específicas para o trabalho em equipe^(9-11)^. O *Canadian Interprofessional Health Collaborative* (CIHC) propõe um quadro de competências, incluindo comunicação interprofissional, trabalho em equipe, liderança colaborativa e atenção centrada no usuário. Tais competências são fundamentais para contextos assistenciais complexos, como os vivenciados pela enfermagem^(10)^.

Reconhecendo a existência de diferentes quadros conceituais consolidados no campo da interprofissionalidade, como os propostos por Barr, pelo *Interprofessional Education Collaborative* e pelo quadro australiano, neste estudo, opta-se por dialogar prioritariamente com o referencial do CIHC, por sua aderência ao contexto formativo e assistencial da enfermagem^(7-11)^.

A integração entre CSEs e o trabalho interprofissional revela-se imprescindível, uma vez que a colaboração transcende dimensões meramente técnicas ou organizacionais, estando intrinsecamente vinculada a processos relacionais e emocionais que garantem a efetividade das interações. Sem as CSEs, o trabalho interprofissional tende a ser superficial, marcado por conflitos e falhas comunicacionais; por outro lado, as CSEs isoladas carecem de impacto coletivo. A articulação entre ambas permite superar modelos fragmentados de atuação, promovendo interdependência, aprendizado mútuo e ambientes éticos e humanizados. No campo da saúde, essa convergência é decisiva para garantir a segurança do usuário dos serviços de saúde e a eficácia das práticas interprofissionais, configurando-se como eixo estruturante da formação e da prática profissional contemporânea^(12,13)^.

Atualmente, muitos enfermeiros trabalham em equipes interprofissionais^(4,14,15)^, tornando a colaboração cada vez mais relevante, sendo a enfermagem a profissão que mais contribui para a articulação entre os profissionais em razão da natureza de seu trabalho centrado no cuidado ao usuário^(16)^. Nesse sentido, a formação em enfermagem tem se apoiado nas Diretrizes Curriculares Nacionais (DCNs), que apresentam competências norteadoras da formação profissional, como liderança, trabalho em equipe, entre outras. A versão mais recente, aprovada pela Resolução CNE/CES nº 573/2018, reforça a importância da formação crítica, ética, humanista e comprometida com a saúde^(17,18)^. Portanto, torna-se essencial articular a formação em prol da interprofissionalidade.

O enfermeiro, como já discutido, é considerado um profissional que articula o cuidado, sendo majoritariamente a categoria que permanece em contato contínuo com os usuários dos serviços de saúde, especialmente em contextos hospitalares e de internação, nos quais está disponível 24 horas por dia. Dessa forma, o uso de práticas colaborativas por essa categoria torna-se essencial para a promoção de ações seguras e de qualidade, ainda que outros profissionais também desempenhem papéis relevantes nesse processo. A presença da interprofissionalidade nas DCNs de enfermagem evidencia a necessidade de superar modelos fragmentados de formação e de atuação. Ao colocar a colaboração entre profissionais como eixo estruturante, as DCNs visam formar enfermeiros mais preparados para o trabalho em equipe, a construção de planos terapêuticos compartilhados e a melhoria contínua da qualidade do cuidado em saúde^(15,19)^.

O referencial utilizado no campo da interprofissionalidade é o de segurança psicológica, pois permite que os membros da equipe se sintam emocionalmente seguros para participar plenamente e colaborar efetivamente. Assim, embora tenha sido constatado que a eficácia da equipe melhora quando os colegas estão emocionalmente seguros uns com os outros^(20)^, poucas iniciativas interprofissionais abordam a dinâmica do desenvolvimento de CSEs na formação para o êxito de práticas colaborativas, principalmente no Brasil. Destarte, embora outra pesquisa não utilize o termo “fator de segurança psicológica” explicitamente, a autora discute aspectos relacionados à cultura do ambiente clínico, à confiança nas relações e ao apoio dos preceptores, que se aproximam da ideia de segurança psicológica (por exemplo: sentir-se acolhido, poder se comunicar sem medo e confiar nos colegas)^(21)^.

Dessa forma, este estudo apresenta o seguinte questionamento: qual a percepção de graduandos de enfermagem sobre como o ensino-aprendizado de CSEs pode impactar o trabalho interprofissional?

A necessidade de investigar a percepção de graduandos de enfermagem no ensino-aprendizado de CSE e o impacto no trabalho interprofissional reside no fato de que, embora essas capacidades sejam reconhecidas como importantes, sua prática eficaz nem sempre é cultivada adequadamente nos currículos tradicionais de formação. Observa-se que programas de ensino se concentram no desenvolvimento de capacidades técnicas específicas e deixam em segundo plano a promoção de CSEs para atuação interprofissional. Ao se debruçar sobre essa lacuna, a presente pesquisa pode estimular reflexões por parte das instituições formadoras na criação de estratégias que integrem o desenvolvimento das CSEs.

Destarte, ao abordar essa temática, a pesquisa pode contribuir para a evolução da participação da enfermagem na dinâmica das equipes interprofissionais, evidenciando que, ao investir no desenvolvimento de CSEs, é possível não apenas melhorar a interação entre os membros da equipe, mas também otimizar os resultados alcançados.

## OBJETIVOS

Analisar a percepção de graduandos de enfermagem sobre as estratégias de ensino-aprendizagem de CSEs e sua influência no trabalho interprofissional.

## MÉTODOS

### Aspectos éticos

O estudo recebeu aprovação do Comitê de Ética em Pesquisa da instituição proponente, conforme Resolução n^o^ 466/12. Os participantes assinaram o Termo de Consentimento Livre e Esclarecido, que assegura o sigilo das informações prestadas.

### Tipo de estudo

Trata-se de estudo exploratório e descritivo, utilizando uma abordagem qualitativa para a coleta de dados. A pesquisa qualitativa, nesse caso, torna-se apropriada, pois possibilita investigar experiências, percepções, comportamentos, culturas e relações sociais, permitindo uma compreensão holística e detalhada das realidades vividas pelos participantes^(22)^. O artigo foi preparado de acordo com as diretrizes do *COnsolidated criteria for REporting Qualitative research* ^(23)^.

### Cenário do estudo

A pesquisa foi conduzida em uma universidade pública brasileira junto ao curso de enfermagem, que oferece dois cursos distintos: bacharelado em enfermagem e bacharelado e licenciatura. Em média, a instituição forma 130 alunos por ano e possui 565 graduandos matriculados no curso, no total, em todos os períodos.

### Fonte de dados

Os participantes deste estudo eram graduandos do último ano dos cursos mencionados que concluíram a disciplina intitulada “Gestão de Enfermagem Hospitalar”. Esses graduandos foram incluídos devido ao conhecimento inicial já explorado na graduação sobre competências, emoções e experiência, considerando que, nesse período, já teriam vivenciado estágios focados em administração e gestão, com ênfase nas habilidades necessárias para a prática de enfermagem. Graduandos que não concluíram a disciplina listada, que estavam com matrícula suspensa ou afastados foram excluídos do estudo.

### Coleta e organização de dados

A coleta de dados ocorreu entre março e setembro de 2023. Os dados foram obtidos por meio da técnica de grupo focal (GF), com as seguintes fases: a primeira consistiu em um questionário sociodemográfico que incluía informações sobre gênero, origem (naturalidade) e tipo de curso de graduação; a segunda utilizou um roteiro com questões norteadoras para discussão grupal, em conformidade com o referencial metodológico descrito por Gatti^(24)^.

Foi empregado o método de amostragem intencional não probabilística. Os participantes foram convidados formalmente, por meio de uma carta convite distribuída após a aula ou enviada eletronicamente, a agendar as reuniões de pesquisa conforme sua conveniência.

Foram realizados nove GFs, sendo oito grupos com a participação de seis graduandos e um grupo com nove graduandos. Todos os grupos foram conduzidos presencialmente e possibilitaram o agrupamento aleatório dos participantes. A coleta foi moderada por um pesquisador com doutorado em ciências e apoiada por um observador, graduado em enfermagem, com mestrado em psicologia e doutorado em ciências. Tanto o moderador quanto o observador são afiliados à mesma instituição educacional, mas não atuam como instrutores, o que ajudou a mitigar qualquer potencial influência de autoridade.

As reuniões do GF foram audiogravadas e duraram, em média, 60 minutos. As perguntas norteadoras para as discussões foram: vocês já tiveram alguma disciplina ou conteúdo que abordasse as CSEs? E trabalho interprofissional? Qual a percepção de vocês sobre a influência de CSEs no trabalho interprofissional? Vocês percebem que a universidade implementou estratégias para desenvolver essas competências em vocês? As perguntas foram elaboradas pelo pesquisador principal, e um diário de campo foi utilizado para documentar as percepções durante e após os GFs, o que auxiliou na análise de dados. A coleta de dados foi considerada completa quando a saturação das informações foi atingida.

### Análise de dados

Para a análise de dados, as discussões dos grupos foram transcritas manualmente pelo pesquisador principal, sem auxílio de *software*, e analisadas por meio da técnica de análise temática indutiva, sustentada pelo referencial teórico de interprofissionalidade e CSE^(6,10)^. Esse processo envolveu transcrição, codificação, agrupamento de tópicos, refinamento de detalhes e uma análise final^(25)^. Esse tipo de análise é considerado ideal, visto que se pretende extrair significados dos participantes. Para manter o anonimato, as falas dos participantes foram identificadas pelo número do GF (GF1, GF2, *etc*.), e as citações foram estruturadas (GF1. Estudante 1...), garantindo que as identidades de todos os indivíduos envolvidos permanecessem confidenciais.

## RESULTADOS

Ao todo, 57 participantes responderam ao questionário, a maioria graduandos: 51 (89,47%) eram do sexo biológico feminino e seis (10,52%), do masculino. Quanto ao curso, 41 (71,92%) eram do bacharelado em enfermagem, e 16 (28,07%), do bacharelado e da licenciatura. Quanto à naturalidade, a maioria era do estado de São Paulo (96%), e os demais eram do estado de Minas Gerais (4%). A partir da análise dos achados, foram construídas duas categorias: “Competências socioemocionais, trabalho interprofissional e o ensino-aprendizagem”; e “Influência das competências socioemocionais no trabalho interprofissional”.

### Competências socioemocionais, trabalho interprofissional e o ensino-aprendizagem

Para os graduandos, as CSEs são associadas ao trabalho em equipe, à liderança compartilhada, à postura, à visibilidade, à adaptabilidade, entre outras:

*Para mim, as competências socioemocionais têm a ver com postura, ética, separar o pessoal do profissional. E a gente, tendo isso, consegue se adaptar na equipe, no setor e ser reconhecido, sabe? Dá visibilidade.* (GF 1. Estudante 3)

*Eu vejo hoje, após muitos estágios, que, claro, a enfermagem não trabalha sozinha, mas para você conseguir isso, você precisa dessas competências, de comunicação clara, respeito, de compartilhar as decisões, ser um líder que aceita ouvir e acatar os outros.* (GF 2. Estudante 4)

Já sobre o trabalho interprofissional, os graduandos associaram a definição à equivalência com o termo “multiprofissional” e evidenciaram os desafios de colocá-lo em prática no estágio:

*Olha, eu já ouvi falar de interprofissional, mas aprendemos como multiprofissional e, pelo que entendi, é diferente, né?! Mas o problema é que, mesmo tendo a oportunidade de termos vários tipos de estágio e em vários lugares, ainda não vi essa colaboração. Ainda fica muito na decisão do médico.* (GF 5. Estudante 9)

*A gente aprendeu, ou melhor, chegou à conclusão no estágio que o trabalho acontece em equipe, precisa ter o multiprofissional, senão o paciente não é atendido de forma integral. Por isso, tem que ter várias profissões atuando.* (GF 7. Estudante 6)

Apesar da importância das CSEs e do trabalho interprofissional, no contexto educacional, o desenvolvimento dessas competências enfrenta uma série de desafios. Para alguns graduandos, as CSEs ainda são vistas como “adicionais” ou secundárias em relação ao conteúdo acadêmico, o que dificulta sua integração nas instituições de ensino.

*Infelizmente, essa parte que comentaram das competências socioemocionais não foi ensinada. Tivemos muito conteúdo sobre humanização, comunicação, mas para com paciente* [...] *nada voltado para nós enquanto enfermeiros, sabe? Até tivemos uma aula de como comunicar más notícias e morte, mas novamente voltado pro paciente.* (GF 4. Estudante 1)

*Vejo que a universidade quer preparar o enfermeiro perfeito robô para o paciente, mas isso das competências socioemocionais é algo secundário. Até falamos de trabalho em equipe, mas, na prática, é cada profissão por si, porque primeiro é os procedimentos e o bem do paciente.* (GF 5. Estudante 8)

### Influência das competências socioemocionais no trabalho interprofissional

As CSEs exercem grande influência no trabalho interprofissional, desempenhando papel fundamental na eficácia, na harmonia e na produtividade das equipes, que envolvem profissionais de diferentes áreas de atuação. Quando bem desenvolvidas, elas contribuem significativamente para o sucesso das equipes interprofissionais, tanto no que diz respeito à qualidade do trabalho realizado quanto ao bem-estar dos profissionais e dos indivíduos:

*Olha, no hospital, foi mais difícil eu ver uma integração e colaboração, mas, na UBS, a equipe era muito unida, sabe? E decidiam tudo em conjunto, e eu percebi, observando, o quanto isso era bom pro cuidado, pro paciente, sabe?* (GF 6. Estudante 2)

*Eu penso que, quando a pessoa tem a capacidade de controlar suas emoções e compreender ser empática com o outro, consegue trabalhar melhor para o prol do paciente.* (GF 9. Estudante 6)

*Se a gente se respeita, consegue trabalhar melhor de forma conjunta, e isso pode ajudar no bem-estar, porque o que eu mais via era conflito e falta de educação, por isso o trabalho não acontecia integrado.* (GF 7. Estudante 3)

Ao mesmo tempo, segundo os graduandos, para trabalhar de maneira interprofissional ou colaborativa, os profissionais devem saber se comunicar de forma eficaz, resolver conflitos, gerenciar o estresse, desenvolver resiliência, empatia e compreensão mútua, que são as CSEs propriamente ditas:

*Eu percebi que, por exemplo, aqui no hospital, claro que tem o trabalho em equipe, mas, para dar certo, precisa respeitar, ser empático, ético. E lidar com pessoas é difícil, mas, para a equipe funcionar, tem que respeitar.* (GF 2. Estudante 5)

*Pessoas são dificeis, né? Eu presenciava muitos conflitos no setor, muito bate-boca, e era comum que um não conversava com o outro e não pedia ajuda só porque não queria ficar com o colega, e isso atrapalha o cuidado, né? Todo mundo vivia estressado, então vejo que a gestão de estresse é importante.* (GF 1. Estudante 4)

*O enfermeiro, ao ter essa habilidade socioemocional para trabalhar, exige flexibilidade e capacidade de adaptação a novas situações e, claro, compreender também o outro.* (GF 5. Estudante 5)

*Por mais que aqui no hospital é um pouco fragmentado, como as pessoas não se falam muito e a decisão ser mais do médico, vejo que é preciso ter um controle emocional, sabe?* (GF 8. Estudante 1)

Destarte, essa influência está atrelada aos bons exemplos de líderes educacionais no campo de estágio e na docência, que contribuíram para o desenvolvimento e a aplicação das CSEs no trabalho interprofissional. Os graduandos atribuíram que esses líderes não devem apenas ser modelos de comportamento emocionalmente inteligentes, mas também atuar para cultivar essas habilidades dentro de suas equipes, pois criam um ambiente de trabalho no qual a empatia, a comunicação aberta e a colaboração são práticas cotidianas, fundamentais para o sucesso das equipes. Além disso, servem como modelo de comportamento emocional, estabelecem espaços seguros para o diálogo, promovem o desenvolvimento profissional e reconhecem a diversidade:

*No hospital onde estou, tem uma enfermeira maravilhosa que se encaixa nessa pergunta. Eu acho que ser emocionalmente preparado tem tudo a ver com o trabalho colaborativo, porque essa enfermeira é um exemplo de como lidar com situações de pressão e conflito. Ela fala bem, sabe? Clara e ela é firme, mas sempre abre as portas para os técnicos aprenderem. Ela incentiva as pessoas a se aprimorarem, então eu vi que ela cria uma cultura de apoio e confiança, não só para os técnicos, mas até os médicos pedem opinião para ela e respeitam, fisios, etc.* (GF 3. Estudante 4)

*Eu via isso um pouco mais na UBS onde estagiei, pois a enfermeira ouvia e parecia que validava essas experiências, o que eu via, e acho que leva a melhores soluções para o caso clínico e maior união no grupo.* (GF 1. Estudante 4)

Isto posto, o trabalho interprofissional nem sempre é fácil de ser colocado em prática. A falta de tempo, de metodologias inovadoras, de recursos e de planejamento de ensino adequado muitas vezes impede que os graduandos tenham clareza da importância de trabalharem de maneira colaborativa e integrada. A divisão do trabalho entre diferentes áreas do conhecimento e a hierarquização das profissões também podem dificultar a construção de um ambiente realmente colaborativo:

*No estágio, eu quase não vi isso de trabalho interprofissional, não. Todo mundo sobrecarregado; o enfermeiro não tinha tempo de promover uma cultura de equipe e trocas, sabe? Era mandar e pronto. Fora que os médicos também não foram formados para isso. Eles acham que mandam.* (GF 6. Estudante 2)

*Quase não tive abordagem disso na graduação. Tinha palestras pontuais de ligas ou projetos de iniciação científica, mas nas aulas, não tínhamos isso claro de como se comportar e como agir com outras pessoas.* (GF 7. Estudante 2)

*Eu discuti isso no estágio com a minha docente* [...] *ela me perguntava como a equipe solucionou isso, mas só vi isso de uma docente, nos outros estágios, não.* (GF 4. Estudante 4)

*Eu vou procurar saber disso de forma individual. Não vi na graduação, mas é difícil porque eu sou uma pessoa que fala, mas tem alguns profissionais que são difíceis e falam: “Isso é da minha área”. Ou então que o médico decide o final.* (GF 3. Estudante 3)

## DISCUSSÃO

Os resultados evidenciaram que os graduandos associaram as CSEs ao trabalho em equipe, à liderança compartilhada, à postura ética e à adaptabilidade, embora apontassem dificuldades na compreensão e na prática do trabalho interprofissional, frequentemente confundido com o multiprofissional e marcado pela centralidade médica. Destacaram ainda a limitada integração das CSEs na formação acadêmica e ressaltaram a influência dessas competências na eficácia e na harmonia das equipes interprofissionais, enfatizando habilidades como empatia, comunicação, gestão de conflitos e controle emocional como indispensáveis para a qualidade do cuidado e o bem-estar dos profissionais.

Destarte, também atribuíram relevância ao papel dos líderes educacionais como modelos de inteligência emocional, capazes de promover ambientes colaborativos. Entretanto, identificaram barreiras estruturais e culturais, como a hierarquização das profissões, a sobrecarga de trabalho e a ausência de metodologias pedagógicas, que dificultam a consolidação do trabalho interprofissional. Como observado no estudo, as CSEs não são aprofundadas no contexto acadêmico, embora objetivem práticas colaborativas.

Vislumbra-se que as universidades muitas vezes priorizam o que pode ser facilmente ensinado, testado e validado dentro de suas metodologias tradicionais, baseadas principalmente em exames, avaliações clínicas e conhecimento técnico. Isso resulta em currículos que favorecem o aprendizado de habilidades diretamente ligadas ao cuidado do usuário dos serviços de saúde, como práticas clínicas e diagnóstico^(26)^. Outro ponto importante é que as CSEs são vistas como algo que os profissionais supostamente desenvolvem por meio de sua experiência e vivência. Essa visão pode levar a falhas no reconhecimento de que essas habilidades podem e devem ser ensinadas, treinadas e avaliadas de maneira mais estruturada dentro do ambiente acadêmico^(27)^.

Outro fator relevante é que a formação acadêmica tem sido tradicionalmente voltada ao desenvolvimento do conhecimento, muitas vezes de forma isolada, sem um foco claro na integração entre diferentes áreas do saber. Em áreas como a saúde, os cursos frequentemente abordam a formação de um profissional que trabalha de maneira individualizada, em vez de preparar os estudantes para o trabalho colaborativo em equipes interprofissionais^(28,29)^, ambiente no qual as CSEs são essenciais.

Além disso, a pressão crescente para que as universidades formem profissionais altamente especializados e capazes de lidar com questões técnicas complexas também tem levado a um descompasso com a necessidade de uma formação mais holística. Nessa linha de pensamento, evidencia-se que as universidades oferecem uma formação baseada em disciplinas e especialidades. Em muitas áreas do conhecimento, os cursos são organizados para capacitar os alunos a atuarem em uma única área de especialização, com ênfase no desenvolvimento de competências técnicas e teóricas relacionadas à sua área específica^(28,30)^.

Dessa forma, observa-se que o currículo acadêmico, em muitas instituições, permanece estruturado sob a lógica de formar profissionais como “especialistas” restritos à sua área de atuação, em vez de prepará-los para serem colaboradores ativos em equipes interprofissionais. Essa perspectiva limitada se reflete nos resultados do estudo, nos quais os graduandos demonstraram confundir o conceito de trabalho interprofissional com o multiprofissional, evidenciando a ausência de uma formação que valorize a integração, a cooperação e a construção coletiva do cuidado.

A infraestrutura das universidades também pode ser um fator limitante. Muitas instituições não possuem recursos suficientes para promover práticas de ensino interprofissional de forma eficaz. Entretanto, a literatura revela que são possíveis atividades práticas e experiências interprofissionais reais, como estágios conjuntos, projetos em equipe e simulações interprofissionais, que promovem eficiência entre diferentes departamentos e programas^(31,32)^. Isso exige investimento especial em tempo, recursos e planejamento institucional, algo que nem sempre é viável em ambientes acadêmicos com restrições orçamentárias ou estruturais.

No entanto, pesquisadores acrescentam que o desconhecimento dos docentes sobre a temática ou até mesmo o dispêndio de tempo e criatividade pode comprometer o desenvolvimento dessas competências nos graduandos de enfermagem^(33)^. Contudo, há relatos de experiências exitosas em âmbito internacional. Como exemplo, os autores evidenciaram um caso de simulação interprofissional, com um paciente padronizado, no qual estudantes de medicina e serviço social trabalharam juntos utilizando a entrevista motivacional. Os resultados indicaram que os graduandos aprenderam como diferentes profissões abordavam questões de saúde e comunicam aos pacientes a importância da colaboração e da necessidade da centralização no usuário dos serviços de saúde, como uma abordagem de cuidado integrada. Assim, esses dados sugerem que simulações interprofissionais padronizadas podem contribuir para um aprendizado eficaz, integral e colaborativo^(34)^.

Ao mesmo tempo, essas lacunas no ensino sobre trabalho interprofissional culminam em falhas na assistência, bem como em confusão entre os termos “interprofissional” e “multiprofissional”, como visto nos achados. No modelo multiprofissional, os profissionais de diferentes áreas trabalham juntos, mas suas atuações tendem a ser mais independentes. Cada profissional, embora faça parte da equipe, realiza suas tarefas de forma isolada, respeitando seus próprios saberes e limites de atuação. A interação entre os membros da equipe pode ocorrer, mas geralmente é menos integrada, com cada um focando em sua área de especialização. Já no trabalho interprofissional, a colaboração é o ponto central. Os profissionais de diferentes áreas trabalham de forma mais integrada, compartilhando conhecimentos, discutindo casos e tomando decisões em conjunto^(35)^.

Dessa forma, evidencia-se a influência das CSEs no trabalho interprofissional, impactando diretamente a eficácia, a harmonia no processo de trabalho e, sobretudo, a assistência integral e qualitativa ao usuário^(15,36)^. Acerca dessa reflexão, uma das formas mais evidentes de influência é por meio da comunicação. Quando um profissional é capaz de expressar suas ideias de maneira clara e assertiva, sem deixar de ser respeitoso e empático, a troca de informações se torna mais eficiente e a compreensão mútua é facilitada. Isso reduz a possibilidade de mal-entendidos e facilita a tomada de decisões coletivas, essenciais para a obtenção de objetivos comuns^(37)^. Isso é particularmente útil em ambientes de ensino, sendo imprescindível o desenvolvimento de variadas estratégias formativas de comunicação voltadas aos profissionais da equipe como um todo.

Além disso, a liderança em equipes interprofissionais é significativamente fortalecida pelo desenvolvimento das CSEs. Conforme observado em nosso estudo, a figura do docente ou preceptor com liderança ativa e colaborativa, pautada na escuta ativa, no apoio mútuo e na valorização de todos os membros da equipe, mostra-se mais eficaz do que modelos autoritários ou baseados na hierarquização profissional. Diferentemente de abordagens verticalizadas, centradas no cargo ou na profissão, a liderança colaborativa promove ambientes de confiança, favorece a corresponsabilidade e potencializa a integração entre diferentes áreas do conhecimento, aspectos indispensáveis para a consolidação do trabalho interprofissional. Ademais, bons líderes e bons exemplos desempenham papel fundamental no sucesso do trabalho interprofissional, pois ajudam a criar um ambiente propício à colaboração, ao respeito mútuo e à eficácia^(38)^.

Por conseguinte, as CSEs são essenciais para o sucesso do trabalho interprofissional, pois ajudam a criar um ambiente de confiança, colaboração e respeito mútuo. Conforme visto em nosso referencial e nos resultados, as CSEs não apenas melhoram a comunicação e a resolução de conflitos, mas também promovem a empatia, a resiliência e a capacidade de adaptação, fundamentais para o funcionamento eficaz de equipes compostas por profissionais de diferentes áreas. Assim, o desenvolvimento dessas habilidades é um investimento que beneficia todos os envolvidos, criando um ambiente mais harmonioso e produtivo^(12,13)^, que deve ser refletido e incorporado nas discussões pedagógicas dos cursos formativos.

Apesar da lacuna acadêmica em aprofundar as CSEs e o trabalho interprofissional, há um movimento crescente nas universidades e nas instituições de ensino para integrar essas competências ao currículo acadêmico. Muitas áreas já formalizaram que um bom profissional não é apenas aquele que possui conhecimento técnico, mas também aquele que sabe se relacionar, comunicar e colaborar com outras pessoas, seja com os colegas de trabalho, seja com os usuários dos serviços de saúde. Cursos de formação mais humanizados, com a inclusão de temas sobre inteligência emocional, comunicação assertiva, resolução de conflitos e trabalho em equipe, estão começando a ser incorporados, especialmente em contextos de saúde e educação^(39)^. No entanto, essa mudança ainda é gradual e depende de maior conscientização sobre a importância dessas habilidades no desempenho profissional.

Assim, os achados reforçam a necessidade de integrar de forma sistemática as CSEs ao processo formativo, articulando-as às competências colaborativas como eixo estruturante da educação e da prática em saúde. Dessa forma, criar um ambiente de sala de aula equilibrado, acolhedor e propício ao desenvolvimento de valores e à construção colaborativa de conhecimentos disciplinares tem se revelado um desafio cada vez maior. Para isso, o professor precisa mobilizar uma ampla gama de competências, como as CSEs^(40)^.

### Limitações do estudo

Esta pesquisa apresenta a limitação de ter sido conduzida unicamente em uma universidade pública, sem incluir análises de outros centros de formação. Além disso, o estudo focou exclusivamente em graduandos de enfermagem do último ano. Portanto, é essencial enfatizar a necessidade de estudos futuros que abranjam outras instituições de ensino, incluindo as privadas, bem como envolvam diversos profissionais e educadores. Isso auxiliará a determinar se há discrepâncias nos achados e em que medida eles podem ser generalizados.

### Contribuições para área da enfermagem, saúde ou políticas públicas

Ao investigar como os graduandos percebem as abordagens utilizadas para desenvolver competências como empatia, comunicação, colaboração, autorregulação emocional e escuta ativa, o estudo oferece subsídios relevantes para aprimorar os currículos dos cursos de graduação, alinhando-os às necessidades contemporâneas do cuidado em saúde, que exigem cada vez mais uma atuação interprofissional eficaz.

Além disso, ao relacionar diretamente o desenvolvimento das CSEs à prática do trabalho em equipe entre diferentes categorias profissionais, o estudo contribui para o fortalecimento da prática colaborativa no contexto do Sistema Único de Saúde e demais cenários de atenção à saúde. A valorização da escuta dos graduandos também favorece a construção de estratégias pedagógicas mais centradas no discente, promovendo o protagonismo do aluno no processo de aprendizagem e o desenvolvimento de uma formação mais humana, crítica e reflexiva. Assim, a pesquisa dialoga com diretrizes nacionais de educação em saúde, como as DCNs, para os cursos de graduação em enfermagem, ao propor caminhos para integrar o ensino técnico-científico com a formação ética e relacional dos futuros profissionais.

## CONSIDERAÇÕES FINAIS

Este estudo evidenciou que, na percepção dos graduandos de enfermagem, as CSEs são necessárias para o trabalho interprofissional, por influenciarem diretamente a comunicação, a resolução de conflitos, a empatia, a compreensão mútua e a integração entre os profissionais de saúde. Dessa forma, responde-se à pergunta de pesquisa ao demonstrar que o ensino-aprendizagem dessas competências impacta de maneira significativa a efetividade e a qualidade do trabalho colaborativo.

Em consonância com o objetivo do estudo, os resultados indicaram que, embora os graduandos reconheçam a relevância das CSEs para a prática interprofissional, as oportunidades de formação dessas competências durante a graduação foram escassas e pouco presentes no currículo formal, ocorrendo de modo pontual e não sistematizado.

Assim, os achados reforçam a necessidade de que as instituições formadoras incorporem, de forma intencional e estruturada, estratégias pedagógicas voltadas ao desenvolvimento das CSEs, visando preparar enfermeiros mais aptos ao trabalho interprofissional e às demandas contemporâneas do cuidado em saúde.

## Data Availability

Os dados de pesquisa estão disponíveis no corpo do artigo.
